# A Phase I Dose-Escalation and Dose-Expansion Study of FCN-437c, a Novel CDK4/6 Inhibitor, in Patients with Advanced Solid Tumors

**DOI:** 10.3390/cancers14204996

**Published:** 2022-10-12

**Authors:** Amita Patnaik, Erika Hamilton, Yan Xing, Drew W. Rasco, Lon Smith, Ya-Li Lee, Steven Fang, Jiao Wei, Ai-Min Hui

**Affiliations:** 1 South Texas Accelerated Research Therapeutics, San Antonio, TX 78229, USA; 2 Breast and Gynecologic Research Program, Sarah Cannon Research Institute, Tennessee Oncology PLLC, Nashville, TN 37203, USA; 3 Department of Medical Oncology & Therapeutics Research, City of Hope National Medical Center, Duarte, CA 91010, USA; 4 Fosun Pharma USA Inc., Lexington, MA 02421, USA

**Keywords:** FCN-437c, CDK4/6 inhibitor, advanced solid tumors, phase I

## Abstract

**Simple Summary:**

Cyclin-dependent kinase 4/6 (CDK4/6) inhibitors have provided clinical benefits for a subset of patients with advanced breast cancer; however, treatment resistance generally emerges over time in patients with breast cancer, and the efficacy of existing CDK4/6 inhibitors in patients with other cancers is modest. The aim of this study was to explore the safety and preliminary antitumor efficacy of a novel, orally available CDK4/6 inhibitor, FCN-437c, in patients with advanced solid tumors. The results demonstrated promising signs of durable tumor response and disease control in this patient population. The safety profile was consistent with that of approved CDK4/6 inhibitors, with no concerning signals in terms of pulmonary, cardiac, or thrombotic risk. The efficacy and safety of FCN-437c merit further study, and this novel agent holds promise as a new alternative treatment for patients with few options.

**Abstract:**

A phase I study evaluated the safety, tolerability, and maximum-tolerated dose (MTD)/recommended phase II dose (RP2D) of FCN-437c, a novel, orally available cyclin-dependent kinase inhibitor (CDK4/6i), in participants with advanced/metastatic solid tumors (aSTs). FCN-437c was escalated from 50 mg (once daily [QD] on days 1–21 of 28-day cycles) to the MTD/RP2D. In the dose-expansion phase, patients with CDK4/6i-treated breast cancer, or *KRAS*-mutant (*KRAS^mut^*) non-small-cell lung cancer (NSCLC) received the MTD. Twenty-two patients were enrolled. The most common tumors in the dose-escalation phase (*n* = 15) were breast, colorectal, and lung (each *n* = 4 [27.3%]). The dose-expansion phase included five (71.4%) patients with breast cancer and two (28.6%) with *KRAS*^mut^ NSCLC. Twenty (90.9%) participants experienced FCN-437c–related adverse events. Dose-limiting toxicities occurred in two (33.3%) participants (200-mg dose, dose-escalation phase): grade 3 neutropenia and grade 4 neutrophil count decreased. Due to toxicities reported at 150 mg QD, the MTD was de-escalated to 100 mg QD. One (4.5%) participant *(KRAS^mut^* NSCLC, 100-mg dose) achieved a partial response lasting 724+ days, and five (22.7%) had stable disease lasting 56+ days. In conclusion, FCN-437c was well tolerated with encouraging signs of antitumor activity and disease control. Further exploration of FCN-437c in aSTs is warranted.

## 1. Introduction

Cyclin-dependent kinases (CDKs) 4 and 6 play a pivotal role in cell-cycle control and progression via phosphorylation and the inactivation of retinoblastoma protein (pRb) [1,2,3]. Loss of cell-cycle control due to aberrant CDK/Rb signaling can occur as a result of mutations in the genes encoding its components or the components of upstream signaling pathways. While such mutations are common in solid tumors, to date, regulatory approval of CDK4/6 inhibitors has only been granted for the treatment of hormone receptor-positive (HR^+^)/human epidermal growth factor receptor 2-negative (HER^–^) breast cancer [1,2,4].

The inhibition of CDK4/6 blocks the CDK/Rb signaling pathway, causing cell-cycle arrest and thereby inhibiting tumor growth and inducing tumor cell apoptosis [1]. First-generation, non-selective pan-CDK inhibitors such as flavopiridol and roscovitine were hampered by limited efficacy and off-target toxicities. Second-generation iterations and beyond have been more selective, targeting specific CDKs toward achieving efficacy with improved safety profiles and reduced off-target effects [3]. To date, three CDK4/6 inhibitors (palbociclib, ribociclib, and abemaciclib) have received regulatory approval for the treatment of HR^+^/HER^–^ breast cancer in combination with aromatase inhibitors (AIs) or fulvestrant, or as a monotherapy (abemaciclib only) in adjuvant and metastatic treatment settings [5,6,7]. The addition of a CDK4/6 inhibitor to endocrine therapy prolonged progression-free survival (PFS) and overall survival (OS) in patients with advanced or metastatic HR^+^/HER2^–^ breast cancer [8,9,10,11]. Based on these results, CDK4/6 inhibitors have become a standard of care for that indication [12,13]. Preclinical and ongoing clinical studies suggest a role for CDK4/6 inhibitors in the treatment of several other solid tumors, including melanoma and non-small cell lung cancer (NSCLC) [1,4]. Although early-stage clinical studies of CDK4/6 inhibitor (palbociclib, abemaciclib) monotherapy demonstrated good safety profiles in small populations of patients with advanced, previously treated NSCLC, the overall antitumor efficacy was limited and generally no better than that achieved with other second-line agents [14]. Studies of CDK4/6 inhibitors combined with immune checkpoint inhibitors and other antitumor agents are assessing whether preclinical signs of synergy translate into therapeutic efficacy in patients with NSCLC [14,15,16].

Despite improved clinical outcomes in advanced/metastatic HR^+^/HER2^–^ breast cancer, the utility of CDK4/6 inhibitors is limited by intrinsic resistance (in approximately 10% of patients) and the acquired resistance that inevitably emerges over time [3,17]. As demonstrated by current regulatory approvals, combination therapy with agents targeting other signaling pathways improves the efficacy of CDK4/6 inhibitors. These combinations were also intended to reduce the risk of adaptive resistance [3]. New CDK4/6 inhibitors are being developed to address drug resistance and to pave the way for re-treatment with other agents in this class after progressive disease (PD) [18].

Brain metastases (BM) develop in an estimated >40% of patients with advanced solid tumors and are particularly prevalent in patients with advanced NSCLC (50%; range 12–65%), breast cancer (25%; range 5–30%), and melanoma (20%; range 12–90%) [19]. BMs are associated with poor prognosis, and until recently, treatment has been palliative [19]. Currently available CDK4/6 inhibitors have proven less effective in patients with primary central nervous system (CNS) and metastatic solid tumors, in most cases due to the limited penetration of the blood–brain barrier (BBB) and rapid elimination from the CNS [20] or (in the case of abemaciclib) uncertain efficacy [21,22,23].

A clear rationale exists for developing new CDK4/6 inhibitors for patients with advanced HR^+^/HER2^−^ breast and other solid tumors (including those with BMs), either alone or in combination with agents targeting other oncogenic pathways, to prevent or overcome treatment resistance. FCN-437c is a novel, selective, potent, and orally active CDK4/6 inhibitor with demonstrated antiproliferative activity in pRb-positive tumor cells derived from a variety of solid tumors [24]. Preclinical findings have shown good BBB penetration and improved potency relative to currently approved CDK4/6 inhibitors and comparable efficacy in combination with AIs or fulvestrant [24]. Further study is warranted to confirm these findings and to determine if they translate into improved clinical outcomes. A recent phase Ia study evaluating the safety and tolerability of FCN-437c reported acceptable safety and a favorable pharmacokinetic (PK) profile in Chinese patients with advanced HR^+^/HER^–^ breast cancer (NCT04488107). While antitumor activity was modest (no complete responses [CRs] or partial responses [PRs]), 60% of patients experienced durable stable disease (SD), suggesting that FCN-437c is capable of inducing disease control [25]. These findings indicate that FCN-437c has potential as a novel and effective targeted therapy for patients with advanced solid tumors. A phase I study was therefore conducted to assess the safety and tolerability of orally administered FCN-437c in patients with advanced solid tumors.

## 2. Materials and Methods

### 2.1. Patients and Study Design

This multicenter, open-label, single-arm dose-escalation and dose-expansion study (NCT03951116) was conducted in the United States. Its primary objectives were to evaluate the safety, tolerability, and maximum tolerated dose (MTD) or recommended phase II dose (RP2D) of orally administered FCN-437c in adult patients (aged ≥ 18 years) with confirmed advanced/metastatic solid tumors whose disease had progressed when receiving currently available therapy or for whom there was no standard therapy. Other key eligibility criteria included no prior treatment with a CDK4/6 inhibitor (except in the case of patients with HR^+^ advanced breast cancer who had received a CDK4/6 inhibitor as standard treatment), Eastern Cooperative Oncology Group performance status of 0 or 1, and evaluable disease per Response Evaluation Criteria in Solid Tumors version 1.1 (RECIST v1.1) [26]. Patients with clinically controlled BMs were eligible for inclusion. A full account of the study eligibility criteria is provided in Supplementary Table S1.

Dose escalation followed a traditional 3 + 3 design, commencing at an FCN-437c dose of 50 mg and escalating in 50-mg increments to 300 mg or until reaching the MTD or RP2D (Supplementary Figure S1). FCN-437c was administered to participants once daily (QD) on day (D)1–D21 of consecutive 28-day cycles. Enrollment into the next higher dose cohort proceeded unless MTD (i.e., the stopping dose) was observed during the first 28-day cycle (cycle [C]1) of the prior dose level. The RP2D would be based on safety data from all participants treated at the MTD, as well as PK and pharmacodynamic data collected across all tested doses.

Dose expansion commenced upon the identification of the MTD, defined as the highest dose level at which ≤1/6 of the participants experienced dose-limiting toxicity (DLT) in C1. Based on preliminary efficacy findings from the dose-escalation phase, only patients with HR^+^/HER2^–^ breast cancer previously treated with a CDK4/6 inhibitor or who had confirmed *KRAS*^mut^ NSCLC were eligible for enrollment into the dose-expansion cohort. The rationale underlying tumor selection for this study is provided in the Supplementary Methods. In both the dose-escalation and dose-expansion cohorts, treatment with FCN-437c continued until PD, intolerable toxicity, withdrawal of consent, or end of study (whichever occurred first), for a maximum of 2 years (unless otherwise approved by the investigator).

The study was conducted in accordance with the principles of the Declaration of Helsinki and Good Clinical Practice guidelines and with the approval of the local Institutional Review Board/Independent Ethics Committee at participating sites. All patients provided informed consent to participate prior to screening and enrollment.

### 2.2. Study Endpoints

Primary endpoints were the incidence, type, and severity of treatment-emergent adverse events (TEAEs), including DLTs, abnormal vital signs, clinical laboratory measurements, and electrocardiograms (ECGs). DLTs were defined as any grade 5 toxicity, FCN-437c–related toxicity (treatment-related adverse event [TRAE]) resulting in treatment discontinuation, TEAEs meeting definition criteria for Hy’s law [27], or any of the following hematologic and nonhematologic toxicities, observed during C1: grade 4 anemia, neutropenia, or thrombocytopenia lasting >7 days, grade ≥3 thrombocytopenia associated with bleeding, grade ≥3 febrile neutropenia, and any grade ≥3 nonhematologic toxicity except for grade 3 nausea or vomiting, grade 3 diarrhea lasting <3 days with adequate supportive care, and grade 3 fatigue lasting <1 week. Secondary endpoints were serum PK parameters for FCN-437c and ORR (based on RECIST v1.1) per investigator assessment.

### 2.3. Study Assessments

TEAEs, including serious TEAEs, were collected throughout the study and for 30 days after the last dose of the study drug, or until all TRAEs had resolved (whichever occurred later). TEAEs were graded using National Cancer Institute Common Terminology Criteria for Adverse Events version 5.0 (NCI CTCAE v5) [28]. Serious TEAEs were adverse events that resulted in death, were life-threatening, required hospitalization or the prolongation of existing hospitalization, resulted in persistent or significant disability/incapacity, were medically important events, or were congenital anomalies/birth defects. The causality of TEAEs was determined by the investigator and categorized as unrelated, possibly related, or probably related to FCN-437c (definitions provided in Supplementary Table S2). TEAEs considered to be either possibly or probably related to FCN-437c are grouped herein as FCN-437c TRAEs.

Clinical laboratory tests, vital signs, physical exams, and ECG results were evaluated during screening and at regular intervals thereafter during treatment, at the end of treatment, and 30 days after the last dose of the study drug. Clinical laboratory parameters included hematology, blood chemistry, coagulation tests, and urinalysis. Blood samples were collected from participants at specific time points to determine the serum concentration of the study drug and/or metabolites using a validated assay for PK analyses. The FCN-437c PK parameters that were evaluated are listed and defined in Supplementary Table S3.

Tumor response was assessed by computed tomography/positron emission tomography/magnetic resonance imaging (according to the standard radiographic procedure for specific tumor sites) per RECIST v1.1 [26] at baseline, after every two cycles of treatment for the first six cycles, and every three cycles thereafter until PD, death, or the investigator’s decision to discontinue study treatment. Best overall response (CR, PR, or SD), ORR (CR + PR according to RECIST v1.1), duration of response (DoR), and DCR (CR + PR + SD) at weeks 6 and 12 were determined.

### 2.4. Statistical Analyses

The sample size was determined according to the number of dose levels evaluated and emerging TRAEs. Approximately 18–36 patients were planned for enrollment in the dose-escalation phase and 6–15 patients in the dose-expansion phase. Safety was assessed in all participants who received at least one dose of FCN-437c. Efficacy was evaluated in those who completed two cycles of treatment and had at least one post-baseline tumor assessment or discontinued early due to clinical progression, PD, lack of efficacy, or death (intention to treat [ITT]). PK parameters were assessed in all participants who received at least one dose of FCN-437c and provided at least one blood sample that was evaluable for PK assessment.

All data were summarized according to FCN-437c dose cohorts. All safety, PK, and efficacy data were summarized using descriptive statistics for continuous variables, and frequencies and percentages for discrete variables. The Kaplan–Meier method was used to analyze time-to-event variables (e.g., DoR). PK parameters were estimated using a noncompartmental method with Phoenix WinNonlin (Certara, formerly Pharsight Corp, Cary, NC, USA). These analyses were conducted using data collected from all enrolled patients up to and including those from the last patient on the last visit (8 December 2021).

## 3. Results

**Figure 1:**
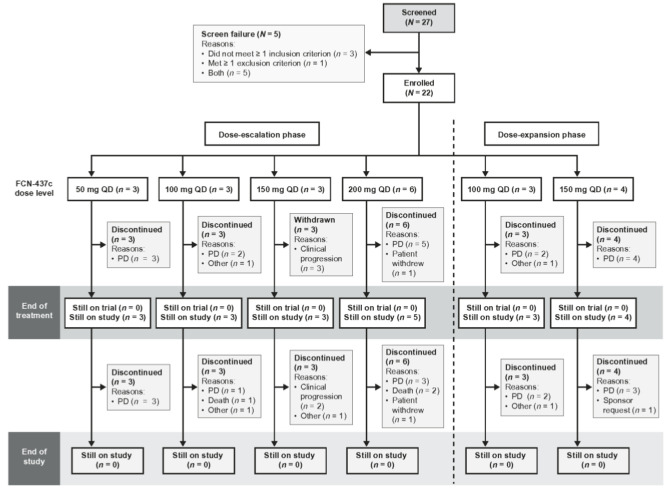
Patient disposition. Abbreviations: PD, progressive disease; QD, once daily.

Twenty-two of the 27 screened patients were enrolled, including 15 in the dose-escalation phase and seven in the dose-expansion phase. At the time of analysis, all 22 patients had received at least one dose of FCN-437c, 15 (68.2%) had completed at least two cycles of study treatment, and all had discontinued study treatment and from the study, primarily due to PD or clinical progression (*n* = 19, 86.4% and *n* = 14, 63.6%, respectively) (Figure 1).

In the dose-escalation phase, participants in the 50-, 100-, 150-, and 200-mg dose cohorts received a median of 2, 4, 1, and 2 cycles of treatment with FCN-437c, respectively, with a range of 1–28 cycles across all dose levels tested. One (33.3%) participant each in the 50-, 100-, and 150-mg dose cohorts received >4 cycles of FCN-437c; three (20.0%) of these participants received ≥10 cycles: one with breast cancer received 21 cycles (50-mg dose cohort), one with *KRAS*^mut^ NSCLC received 28 cycles (100-mg cohort), and another with breast cancer received 10 cycles (150-mg cohort). In the dose-expansion phase, no participant received >2 cycles of study treatment (two participants received 1 cycle and five received 2 cycles). The safety analysis, ITT, and PK sets included all 22 enrolled patients.

### 3.1. Patient Demographics and Baseline Characteristics

The entire study cohort had a median age of 64 years (range, 39–88 years) and was predominantly female (*n* = 14, 63.6%). The most common solid tumors in the dose-escalation phase were breast, colorectal, and NSCLC (*n* = 4 for each). Five (71.4%) participants in the dose-expansion phase had breast cancer and two (28.6%) had *KRAS*^mut^ NSCLC. All nine patients with HR^+^ breast cancer had received prior therapy with a CDK4/6 inhibitor: four in the dose-escalation phase and five in the dose-expansion phase. Among those in the dose-escalation phase, two had received abemaciclib and two had received palbociclib. Three of the five patients in the dose-expansion phase had received only palbociclib, one had received only abemaciclib, and one had received both palbociclib and abemaciclib. Across both study phases, all but one patient had stage IV disease; the exception was a participant in the 200-mg dose-escalation group who had stage III disease (Table 1).

### 3.2. Dose-Limiting Toxicities

Two (33.3%) participants in the dose-escalation phase (200-mg dose cohort) reported DLTs: grade 3 neutropenia (*n* = 1, 16.7%) and grade 4 neutrophil count decreased (*n* = 1, 16.7%); both events resolved, and both were assessed as being related to FCN-437c. No cases of febrile neutropenia were reported. The MTD was initially determined to be 150 mg QD, and four participants were subsequently treated at that dose in the expansion phase. Only one of these participants completed the first cycle of treatment, and the other three discontinued due to toxicity; therefore, the Safety Review Committee determined that the MTD should be de-escalated to 100 mg QD. Three participants were subsequently treated at the 100-mg dose in the dose-expansion phase with no significant toxicity.

### 3.3. Safety and Tolerability

All participants reported at least one TEAE and 16 (72.7%) experienced at least one grade ≥3 TEAE (Table 2). The most frequently observed TEAEs were neutrophil count decreased (*n* = 8, 36.4%), white blood cell count (WBC) decreased (*n* = 7, 31.8%), and fatigue (*n* = 6, 27.3%). The most common grade ≥ 3 TEAEs were neutrophil count decreased, WBC decreased (*n* = 6, 27.3% for each), and anemia (*n* = 2, 9.1%); all others were reported in one (4.5%) participant each. No venous or arterial thromboembolic events were reported.

Twenty (90.9%) participants experienced at least one TRAE, most of which were hematologic (Supplementary Table S4). Two participants in the 100-mg dose cohort of the dose-expansion phase did not experience any FCN-437c TRAEs. The most commonly occurring TRAEs across all dose cohorts were neutrophil count decreased (*n* = 9, 40.9%), WBC decreased (*n* = 8, 36.4%), fatigue (*n* = 6, 27.3%), and lymphocyte count decreased (*n* = 5, 22.7%), each accounting for all occurrences of these particular TEAEs. No FCN-437c–related treatment or study discontinuations or deaths were reported.

Two (9.1%) participants, both in the dose-escalation phase, experienced a serious adverse event (SAE): grade 3 abdominal wall abscess (100-mg dose cohort) and grade 3 sepsis (200-mg dose cohort). Both cases were resolved, and neither was considered related to FCN-437c. TEAEs and TRAEs led to dose interruption/modification in 16 (72.1%) and 13 (59.1%) participants, respectively. The most frequent TRAEs leading to dose interruption/modification were neutrophil count decreased (*n* = 5, 22.7%; grade 3 in four participants and grade 4 in one participant), WBC decreased (*n* = 4, 18.2%; grade 2 in one participant, grade 3 in two participants, and grade 4 in one participant), and platelet count decreased (*n* = 3, 13.6%; all grade 2) (Supplementary Table S5). Five deaths occurred during the study; four were due to PD and one was categorized as being due to “other” (participant died in hospice).

### 3.4. Other Safety Parameters

Most mean values for chemistry and hematology results were within normal ranges; those that fell outside of normal ranges were not considered to be clinically significant. Mean hematologic values decreased after the start of the study treatment. The mean values of the coagulation and urinalysis results, as well as those for vital signs, were within normal ranges, with no apparent trends in mean changes from baseline. In general, abnormal physical examination findings were not considered to be clinically significant. The exceptions were four participants who reported abnormal dermatological findings; two each from the dose-escalation and dose-expansion phases. In the dose-escalation phase, one participant had grade 1 abrasion (100-mg dose cohort) and another had grade 1 skin hyperpigmentation (150-mg dose cohort). In the dose-escalation phase, one participant had a grade 2 bruise following a fall, and another had grade 3 macropapular rash (both in the 150-mg dose cohort). Only the grade 3 macropapular rash was considered possibly related to FCN-437c. Most participants had abnormal, but not clinically significant, ECG findings at baseline and throughout the study. Only one participant, a 66-year-old female with stage IV adenocarcinoma of the liver and bile duct (200-mg dose cohort in the dose-escalation phase), had a clinically significant ECG abnormality: severe (grade 3) supraventricular tachycardia, low QRS voltage in limb leads, pattern consistent with pulmonary disease, and moderate ST depression at an unscheduled visit. No dose alterations were needed, and the condition, which was not considered to be related to FCN-437c, was ultimately resolved without the need for treatment.

### 3.5. Pharmacokinetics

**Figure 2:**
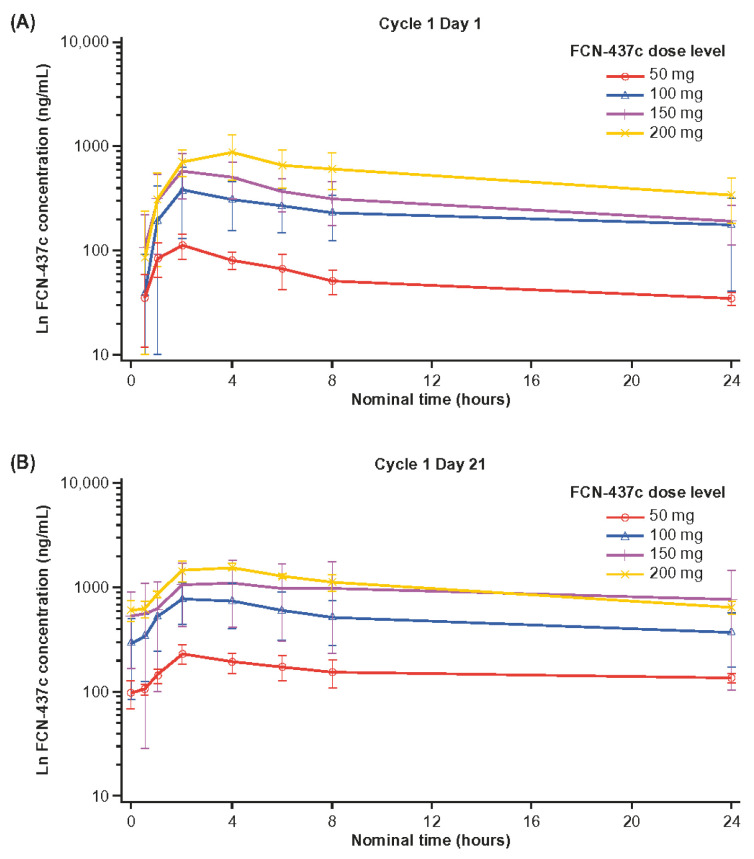
Semi-log concentration-time plot of mean FCN-437c concentration over 24 h post dose for all dose levels at Cycle 1 Day 1 (**A**) and Cycle 1 Day 21 (**B**) The 100- and 150-mg dose levels include both the dose-escalation and dose-expansion cohorts. Error bars are standard deviation.

PK data are provided in Figure 2 and Supplementary Table S6. FCN-437c was rapidly absorbed, with the time to maximum plasma concentration (C_max_) of FCN-437c ranging between 2 and 4 h post-dose across dose cohorts. Overall exposure increased with increasing dosage, and no plateau was reached at any dose. For the C_max_ and area under the time–concentration curve (AUC) between time 0 and 24 h post-dose (AUC_0-24_), a two-fold increase in the dose from 50 to 100 mg/day resulted in an approximately four-fold increase in exposure, except for C1D21 when AUC_0–24_ increased three-fold. Exposure increased dose proportionately for the dose range of 100–200 mg/day on both C1D1 and C1D21. The AUC extrapolated between time 0 and infinity on C1D1 was close to dose-proportional over the dose range of 50–200 mg/day.

The trough concentration of FCN-437c was similar for most dose levels between C1D14 and C1D21, indicating that steady state was likely achieved by C1D14. On C1D21, FCN-437c accumulation was about two- to three-fold in all except the 200-mg dose cohort, in which a slightly lower accumulation was observed (approximately 1.5-fold). The elimination half-life of FCN-437c ranged from 14.5 to 20.5 h across all doses on C1D1 and from 14.9 to 45.5 h on C1D21.

### 3.6. Efficacy

**Figure 3:**
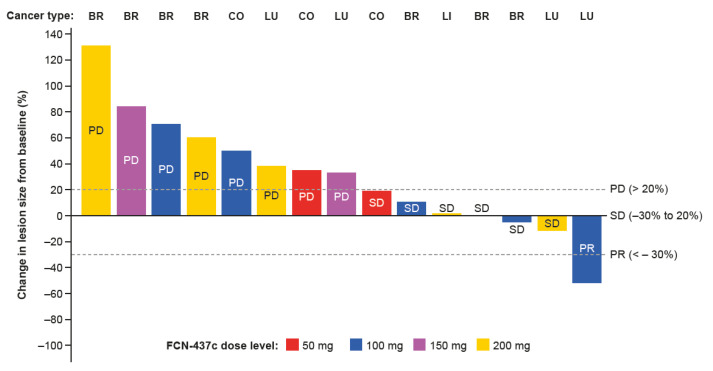
Waterfall plot showing best percentage change from baseline in lesion size and best response for target lesions of all evaluable patients, with tumor type. Abbreviations: BR, breast cancer; CO, colorectal cancer; LI, liver cancer; LU, lung cancer; PD, progressive disease; PR, partial response; SD, stable disease.

A summary of the efficacy parameters is provided in Table 3. In the dose-escalation phase, one participant with *KRAS*^mut^ NSCLC (100-mg dose cohort) achieved the best overall objective response of confirmed PR, and four participants achieved SD, two with breast cancer (one each in the 50- and 150-mg dose cohorts), one with cancer of the liver and bile duct (200-mg dose cohort), and one with *KRAS*^mut^ NSCLC (200-mg dose cohort) (Figure 3, Table 3). In the dose-expansion phase, one participant with breast cancer (100-mg dose cohort) achieved SD. The ORR and DCR (at 12 weeks) were thus 4.5% and 27.3%, respectively; the median DoR was not reached (Table 3). Ten (45.5%) participants had a best response of PD. Six participants (four in the dose-escalation phase and two in the dose-expansion phase) were not evaluable for efficacy. Among the patients with breast cancer who achieved SD, two had previously received palbociclib (one in the 150-mg dose cohort and one in the 100-mg dose cohort of the dose-expansion phase), and one had previously received abemaciclib (50-mg dose cohort). The participant who achieved PR was a 70-year-old female with stage IV, heavily pretreated, *KRAS*^mut^ NSCLC that was progressing at the time of enrollment. This participant received 28 cycles of FCN-437c treatment and at the time of analysis had a DoR of 724 days and PFS of 827 days. This participant discontinued from the dose-escalation study and has continued to receive FCN-437c through the investigator’s extended access program.

## 4. Discussion

This phase I, open-label study demonstrated that FCN-437c delivered orally QD for 21 days in continuous 28-day cycles was generally well tolerated in participants with advanced or metastatic solid tumors that had progressed on available therapies or for which there was no standard therapy. There were no unexpected safety signals, TEAEs leading to death, FCN-437c–related SAEs, or TEAEs leading to treatment discontinuation or study withdrawal. Two (33.3%) participants in the 200-mg dose cohort (dose-escalation phase) reported DLTs: the grade 3 neutropenia and grade 4 neutrophil count decreased. Although the MTD of FCN-437c was initially determined as 150 mg QD, the dose was later de-escalated to 100 mg QD by the Safety Review Committee based on a review of the tolerability data.

A 70-year-old female with heavily pretreated, progressive (at study entry) *KRAS*^mut^ NSCLC achieved a durable objective response of confirmed PR that was ongoing at study closure and was thus allowed to continue to receive FCN-437c. Five (22.7%) participants across both study phases had the best response of SD, indicating the potential for FCN-437c to confer disease control. Experience with other CDK4/6 inhibitors suggests that promising efficacy signals such as these might be enhanced through rational combinations with AIs, fulvestrant, or antitumor agents with different targets/mechanisms of action (including radiation therapy) suited to the tumor of interest [3,8,29,30,31]. Evidence from other studies suggests that patients who have previously progressed while receiving a CDK4/6 inhibitor can benefit from re-treatment with a CDK4/6 inhibitor or switching to a different one [18,32,33]. In the present study, among five participants with advanced breast cancer previously treated with a CDK4/6 inhibitor in the dose-expansion phase, the only participant evaluable for response (25.0%), who had previously received palbociclib, achieved SD.

Neutrophil count decreased and WBC count decreased were the most frequently reported TRAEs and grade ≥3 TEAEs. This is consistent with findings from an ongoing multicenter phase Ia FCN-437c monotherapy study in China that is enrolling women with HR^+^/HER2^–^ advanced breast cancer (NCT04488107) [25], as well as single-agent data reported for approved CDK4/6 inhibitors [29,34,35]. The safety profile of FCN-437c compared favorably with abemaciclib, with participants experiencing the most common TEAEs (neutropenia, thrombocytopenia, fatigue, diarrhea) at lower rates with reduced severity and lower rates of vomiting [35]. Class effects otherwise reported for approved CDK inhibitors, such as venous thromboembolic events (including pulmonary and deep vein thrombosis) and severe pneumonitis/interstitial lung disease [36,37,38] were not observed during the present study.

The PK data may support the use of FCN-437c doses of 150–200 mg QD in phase III trials, depending on the tumor type. In a phase I trial of abemaciclib, the PK and pharmacodynamic data were generally comparable for 150 mg administered twice daily (BID) and 200 mg BID, and the MTD was set at 200 mg BID. However, it was noted that in certain clinical contexts the lower dose could be initiated to limit toxicity, with the consideration of escalating to 200 mg BID on an individual basis depending on response/toxicity [21]. In subsequent phase III trials, abemaciclib was initiated at 200 mg BID for patients with *KRAS*^mut^ NSCLC [39] and at 150 mg BID for patients with *ERBB2*-negative breast cancer [11]. Conversely, with FCN-437c, some patients may be able to tolerate higher doses for the sake of improved efficacy.

Promising preclinical trial data on FCN-437c regarding BBB penetration [24] could not be assessed in this small study due to the lack of patients with BMs at the study entry. Assessing whether and how this property of FCN-437c might impact tumoral and overall clinical response will require study in larger populations that would include patients with BM or CNS involvement. The present findings support further study of FCN-437c, a novel CDK4/6 inhibitor with potential as a treatment for patients with advanced solid tumors. Such studies warrant larger cohorts of patients with advanced solid tumors treated with FCN-437c monotherapy, in combination with AIs or fulvestrant (as indicated), or other anticancer agents such as PI3K or mTOR inhibitors. To this end, two ongoing phase II studies in China are evaluating FCN-437c alone versus in combination with letrozole (NCT04488107) [40], or in combination with fulvestrant or letrozole plus goserelin (NCT05004142) [41] in patients with HR^+^/HER2^–^ advanced breast cancer; in addition, two ongoing phase III trials are evaluating FCN-437c versus placebo in combination with letrozole or anastrozole with or without goserelin (NCT05439499) or FCN-437c versus placebo in combination with fulvestrant with or without goserelin (NCT05438810) in patients with HR^+^/HER2^–^ advanced breast cancer. Preclinical and clinical data now support further evaluation of FCN-437c in other indications, for example in patients with *KRAS*^mut^ solid tumors and in patients with CNS involvement. The combination with other targeted therapies may be explored to overcome or mitigate the development of CDK4/6 inhibitor resistance. In order to enrich for patients likely to benefit from CDK4/6 inhibitors, translational studies have been nested within FCN-437c trials in order to identify biomarkers predictive of response (e.g., high cyclin-D1 levels in patients with estrogen-receptor-positive luminal breast cancer [42]) or the likelihood of developing resistance to CDK4/6 inhibitors (e.g., evidence of a functional loss of pRb at baseline, those with metastatic breast cancer and high levels of cyclin-E1 expression or *myc* alteration, high serum thymidine kinase 1, and aberrations in components of tyrosine kinase receptor signaling) [43,44].

The strength of the present study lies in the demonstrated tolerability and safety profile of FCN-437c, an orally available agent with promising signs of disease control. The findings are limited by the phase I, single-arm study design and small sample size, which prevented the assessment of preclinical leads regarding FCN-437c synergy with hormonal therapy and potential to penetrate the BBB.

## 5. Conclusions

FCN-437c was generally well-tolerated at the MTD, with no SAEs related to FCN-437c or TEAEs leading to death. The MTD was ultimately identified as 100 mg QD. Encouraging signs of disease stabilization with FCN-437c monotherapy were observed, notably in a participant with heavily pretreated NSCLC (treatment ongoing). This promising efficacy signal is consistent with preclinical data that suggest a synthetically lethal relationship between *KRAS* oncogenes and loss of CDK4 or CDK6, leading to the consideration of CDK4/6 targeting as a treatment strategy for *KRAS*^mut^ tumors [45,46]. These data support further exploration of FCN-437c in larger cohorts of patients with advanced solid tumors, in combination with endocrine therapy (e.g., AIs, fulvestrant) or antitumor agents with different targets/mechanisms of action appropriate to the tumor of interest.

## Figures and Tables

**Table 1:** Summary of patient baseline demographic and baseline disease characteristics.

Characteristic *n* (%), Unless Otherwise Stated	FCN-437c Dose-Escalation Phase *N* = 15				FCN-437c Dose-Expansion Phase *N* = 7		Total *N* = 22
	50 mg *n* = 3	100 mg *n* = 3	150 mg *n* = 3	200 mg *n* = 6	100 mg *n* = 3	150 mg *n* = 4	
**Age, years**							
Median (range)	64.0 (52–71)	70.0 (69–77)	64.0 (51–64)	62.5 (45–88)	56.0 (39–67)	59.0 (54–77)	64.0 (39–88)
<65 years	2 (66.7)	0	3 (100)	3 (50.0)	2 (66.7)	3 (75.0)	13 (59.1)
≥65 years	1 33.37)	3 (100)	0	3 (50.0)	1 (33.3)	1 (25.0)	9 (40.9)
Sex							
Female	1 (33.3)	1 (33.3)	2 (66.7)	5 (83.3)	3 (100)	2 (50.0)	14 (63.6)
Male	2 (66.7)	2 (66.7)	1 (33.3)	1 (16.7)	0	2 (50.0)	8 (36.4)
**Ethnicity**							
Hispanic or Latino	1 (33.3)	0	2 (66.7)	1 (16.7)	1 (33.3)	1 (25.0)	6 (27.3)
Not Hispanic or Latino	2 (66.7)	3 (100)	1 (33.3)	4 (66.7)	1 (33.3)	3 (75.0)	14 (63.6)
Not reported	0	0	0	1 (16.7)	1 (33.3)	0	2 (9.1)
**Primary tumor site**							
Breast	1 (3.33)	0	1 (3.33)	2 (3.33)	3 (100)	2 (50.0)	9 (40.9)
Uterine ^a^	0	0	0	1 (16.7)	0	0	1 (4.5)
Colorectal	2 (66.7)	1 (33.3)	1 (33.3)	0	0	0	4 (18.2)
Liver and bile duct	0	0	0	1 (16.7)	0	0	1 (4.5)
Pancreatic ^b^	0	0	1 (33.3)	0	0	0	1 (4.5)
Lung	0	2 (66.7)	0	2 (3.33)	0	2 (50.0)	6 (27.3)
**Tumor stage**							
III	0	0	0	1 (16.7)	0	0	1 (4.5)
IV	3 (100)	3 (100)	3 (100)	5 (83.3)	3 (100)	4 (100)	21 (95.5)

^a^ Corpus uteri, including endometrial. ^b^ Pancreas, exocrine, and endocrine.

**Table 2:** Summary of treatment-emergent adverse events (TEAEs) by dose group and for all patients (safety population, *N* = 22), and summary of the most commonly occurring (in ≥10% of the total population) TEAEs.

TEAEs, *n* (%)	FCN-437c Dose-Escalation Phase (*N* = 15)				FCN-437c Dose-Expansion Phase (*N* = 7)		Total (*N* = 22)
	50 mg (*n* = 3)	100 mg (*n* = 3)	150 mg (*n* = 3)	200 mg (*n* = 6)	100 mg (*n* = 3)	150 mg (*n* = 4)	
Any TEAE	3 (100)	3 (100)	3 (100)	6 (100)	3 (100)	4 (100)	22 (100)
Related to FCN-437c ^a^	3 (100)	3 (100)	3 (100)	6 (100)	1 (33.3)	4 (100)	20 (90.9)
Grade ≥ 3	2 (66.7)	2 (66.7)	3 (100)	4 (66.7)	1 (33.3)	4 (100)	16 (72.7)
SAEs	0	1 (33.3)	0	1 (16.7)	0	0	2 (9.1)
Leading to dose interruption	1 (33.3)	1 (33.3)	3 (100)	4 (66.7)	0	2 (50.0)	11 (50.0)
Leading to dose modification ^b^	1 (33.3)	0	1 (33.3)	1 (16.7)	0	1 (25.0)	4 (18.2)
**TEAEs occurring in ≥10% of the total population**							
Neutrophil count decreased	1 (33.3)	1 (33.3)	0	3 (50.0)	0	3 (75.0)	8 (36.4)
WBC decreased	0	1 (33.3)	2 (66.7)	2 (33.3)	0	2 (50.0)	7 (31.8)
Fatigue	1 (33.3)	0	0	4 (66.7)	0	1 (25.0)	6 (27.3)
Lymphocyte decreased	0	0	0	2 (33.3)	1 (33.3)	2 (50.0)	5 (22.7)
Nausea	0	1 (33.3)	1 (33.3)	2 (33.3)	0	1 (25.0)	5 (22.7)
Diarrhea	0	1 (33.3)	0	2 (33.3)	0	1 (25.0)	4 (18.2)
Dyspnea	1 (33.3)	0	0	1 (16.7)	1 (33.3)	1 (25.0)	4 (18.2)
Platelet count decreased	0	0	1 (33.3)	1 (16.7)	0	2 (50.0)	4 (18.2)
Anemia	1 (33.3)	0	0	2 (33.3)	0	0	3 (13.6)
Cough	1 (33.3)	0	1 (33.3)	0	0	1 (25.0)	3 (13.6)
Dehydration	0	0	0	2 (33.3)	0	1 (25.0)	3 (13.6)
Fall	1 (33.3)	1 (33.3)	0	0	0	1 (25.0)	3 (13.6)
Upper respiratory tract infection	1 (33.3)	0	2 (66.7)	0	0	0	3 (13.6)

Abbreviations: SAE, serious adverse event; TEAE, treatment-emergent adverse event; WBC, white blood cell. ^a^ Considered by the investigator to be possibly or probably related to FCN-437c. ^b^ All were dose reductions except for one patient in the 50-mg dose cohort, for whom the dose was increased.

**Table 3:** Summary of efficacy parameters (ITT population, *N* = 22).

	Dose-Escalation Phase (*N* = 15)				Dose-Expansion Phase (*N* = 7)		Total (*N* = 22)
	50 mg (*n* = 3)	100 mg (*n* = 3)	150 mg (*n* = 3)	200 mg (*n* = 6)	100 mg (*n* = 3)	150 mg (*n* = 4)	
Best overall response, *n* (%)	3 (100)	3 (100)	3 (100)	6 (100)	1 (33.3)	4 (100)	20 (90.9)
PR (confirmed)	0	1 (33.3)	0	0	0	0	1 (4.5)
SD	1 (33.3)	0	1 (33.3)	2 (33.3)	1 (33.3)	0	5 (22.7)
PD	2 (66.7)	1 (33.3)	0	3 (50.0)	2 (66.7)	2 (50.0)	10 (45.5)
Not evaluable	0	1 (33.3)	2 (66.7)	1 (16.7)	0	2 (50.0)	6 (27.3)
ORR, ^a^ % (95% CI)	0	33.3 (0.8–90.6)	0	0	0	0	4.5 (0.1–22.8)
DCR ^b^ at 12 weeks, *n*	1	1	1	2	1	0	6
% (95% CI)	33.3 (0.8–90.6)	33.3 (0.8–90.6)	33.3 (0.8–90.6)	33.3 (4.3–77.7)	33.3 (0.8–90.6)	0	27.3 (10.7–50.2)
DoR, median (95% CI)	NR	NR	NR	NR	NR	NR	NR
Duration of SD (days), median (95% C)	57 (49–NR)	NR (112–NR)	NR (NR–NR)	69 (56–NR)	56 (54–NR)	56 (51–NR)	112 (56–NR)

Abbreviations: CI, confidence interval; CR, complete response; DCR, disease control rate; DoR, duration of response; ITT, intention to treat; ORR, objective response rate; PD, progressive disease; PR, partial response; RECIST v1.1, Response Evaluation Criteria in Solid Tumors version 1.1. ^a^ ORR = CR + PR. ^b^ DCR = CR + PR + SD according to RECIST v1.1 [26].

## Data Availability

Additional data are available in the supplementary material. Datasets are available upon reasonable request from the corresponding author.

## Supplementary Materials

The following supporting information can be downloaded at: https://www.mdpi.com/article/10.3390/cancers14204996/s1, Table S1: Study eligibility criteria; Figure S1: Phase I dose-escalation and dose-expansion study design; Table S2: Causality of adverse events; Table S3: List of pharmacokinetic parameters evaluated and their definitions; Table S4: Most frequently observed (in ≥2 participants in the safety analysis set) TEAEs considered by the investigator to be possibly or probably related to FCN-437c and SAEs by dose group and for all participants (safety population, *N* = 22); Table S5: TEAEs leading to dose interruption that were considered by the investigator to be possibly or probably related to FCN-437c (TRAEs), by dose group and for all participants (safety population, *N* = 22); Table S6: Summary of key FCN-437c pharmacokinetic parameters for Cycle 1 Day 1 and Cycle 1 Day 21 by dose group.
