# Supplementary material for: A Phase I Dose-Escalation and Dose-Expansion Study of FCN-437c, a Novel CDK4/6 Inhibitor, in Patients with Advanced Solid Tumors
Source: Cancers (Basel). 2022 Oct 12;14(20):4996. doi: 10.3390/cancers14204996 (PMC9599640; doi:10.3390/cancers14204996)
Supplement: Supplementary file 1 [file cancers-14-04996-s001.zip › cancers-1927580-supplementary.pdf]

Supplement

Supplementary Methods

Table S1. Study eligibility criteria.

**Inclusion criteria**
▪ Provided written informed consent prior to any study-specific procedures
▪ Male or female subject ≥18 years.
▪ Histologically/cytologically confirmed, unresectable locally advanced or metastatic solid tumors that are refractory to standard therapy or for which no standard therapy exists. Note for subjects with NSCLC and subjects with tumors showing activating *ALK* translocation or *EGFR* mutations, they must have been treated and failed appropriate targeted treatment. Subjects enrolled in cohort expansion at the MTD should have specific tumor types as below:<br>− *KRAS*^{mut} NSCLC confirmed by a documented historical report<br>− Breast cancer previously treated with a CDK4/6 inhibitor
▪ All subjects should have evaluable disease as per RECIST v1.1 [1]. Subjects enrolled in cohort expansion at the MTD should have measurable disease (presence of at least one measurable lesion) as per RECIST v1.1.
▪ ECOG PS ≤1.
▪ Life expectancy of ≥3 months
▪ Subjects with CNS metastases were eligible if clinically controlled that is defined as surgical excision and/or radiation therapy followed by 3 weeks of stable neurologic function and no evidence of CNS disease progression, as determined by contrast-enhanced computed tomography and nuclear magnetic resonance imaging ≤3 weeks prior to the first dose of study drug
▪ Adequate organ function, including the following:<br>− Bone marrow reserve: ANC ≥1.5×10^9/L; platelet count ≥100×10^9/L; hemoglobin ≥9 g/dL or ≥5.6 mmol/L (initiation of transfusions of blood and blood products is not allowed ≤2 weeks before first dose of FCN-437c)<br>− Hepatic: total bilirubin ≤1.5×ULN, AST and/or ALT ≤2.5×ULN (<5×ULN if liver metastases present)<br>− Renal: serum creatinine ≤1.5×ULN or estimated creatinine clearance ≥45 mL/min based on the Cockcroft-Gault equation<br>− Coagulation: prothrombin time/international normalized ratio or activated partial thromboplastin time ≤1.5×ULN
▪ Subjects were able to swallow capsules
▪ Subjects (femalesof child-bearing potential and males) should be willing to use viable contraception method that is deemed effective by the investigator throughout the treatment period and for ≥3 months following the last dose of study drug. Postmenopausal women must have been amenorrheic for ≥12 months to be considered of non-childbearing potential

Exclusion criteria
▪ Females during pregnancy or breastfeeding
▪ Subjects on any anticancer therapy approved or experimental, including chemotherapy, immune therapy, radiation therapy, hormonal therapy (exceptions: hormone-replacement therapy, testosterone or oral contraceptives), biologic therapy, ≤3 weeks (or 5 half-lives, whichever is shorter) prior to initiation of study treatment. Note: subjects should be recovered to baseline from any treatment-related toxicity, except for residual alopecia
▪ Subjects who had prior treatment with a CDK4/6 inhibitor (with the exception of hormone receptor-positive breast cancer subjects who may have received CDK4/6 inhibitor as a standard treatment, which was allowed)
▪ Subjects with a history of gastric bypass surgery or banding procedure
▪ Subjects who have had major surgery ≤28 days from screening or those who have undergone organ transplant surgery
▪ Active HBV or HCV infection. HBV carriers without active disease (HBV DNA titer <1,000 cps/mL or 200 IU/mL), or cured HCV (negative HCV RNA test) could be enrolled. Subjects with controlled human immunodeficiency virus disease may be eligible
▪ Subjects with a concomitant medical condition that would increase the risk of toxicity, in the opinion of the investigator or Sponsor
▪ Unresolved toxicities (other than alopecia) from previous anticancer therapy defined as toxicities not resolved to NCI CTCAE v5.0 [2], grade ≤1
▪ Subjects who have had severe infection ≤4 weeks or signs and symptoms of any active infection ≤2 weeks prior to the first dose administration
▪ Severe or uncontrolled cardiac disease requiring treatment, congestive heart failure NYHA class III or IV, unstable angina pectoris even if medically controlled, history of myocardial infarction during the last 3 months, serious arrhythmias requiring medication (with exception of atrial fibrillation or paroxysmal supraventricular tachycardia)
▪ A resting ECG with QTcF ≥470 ms or the subject has a congenital prolonged QT syndrome
▪ Taking a prohibited concomitant medication or inability to follow concomitant medications guidelines
▪ Any other serious underlying medical (eg, uncontrolled diabetes mellitus, uncontrolled hypertension, active uncontrolled infection, active gastric ulcer, uncontrolled seizures, severe hearing impairment, cerebrovascular incidents, gastrointestinal bleeding, severe signs and symptoms of coagulation and clotting disorders, cardiac conditions), psychiatric, psychological, familial, or geographical condition that, in the judgment of the investigator, may interfere with the planned staging, treatment and follow-up, affect subject compliance or place them subjectat high risk of treatment-related complications

Abbreviations: ALK, anaplastic lymphoma kinase; ALT, alanine aminotransferase; ANC, absolute neutrophil count; AST, aspartate aminotransferase; CDK4/6, cyclin-dependent kinases 4/6; CNS, central nervous system; DNA, deoxyribonucleic acid; ECG, electrocardiogram; ECOG PS, Eastern Cooperative Oncology group performance status; EGFR, epidermal growth factor receptor; HBV, hepatitis B virus; HCV, hepatitis C virus; MTD, maximum tolerated dose; NCI CTCAE v5.0, National Cancer Institute Common Terminology Criteria in Solid Tumors version 5.0; NSCLC, non-small cell lung cancer; NYHA, New York Heart Association; QTcF, QT interval corrected using Fridericia's formula; RECIST v1.1, Response Evaluation Criteria in Solid Tumors version 1.1; RNA, ribonucleic acid; ULN, upper limit of normal.

Study Design

Figure S1. Phase I dose-escalation and dose-expansion study design. Abbreviations: DLT, dose-limiting toxicity; *KRAS*^{mut}, mutation in the gene encoding K-Ras protein; MTD, maximum- tolerated dose; NSCLC, non-small cell lung cancer; PK, pharmacokinetic; QD, once daily; RP2D, recommended phase II dose.

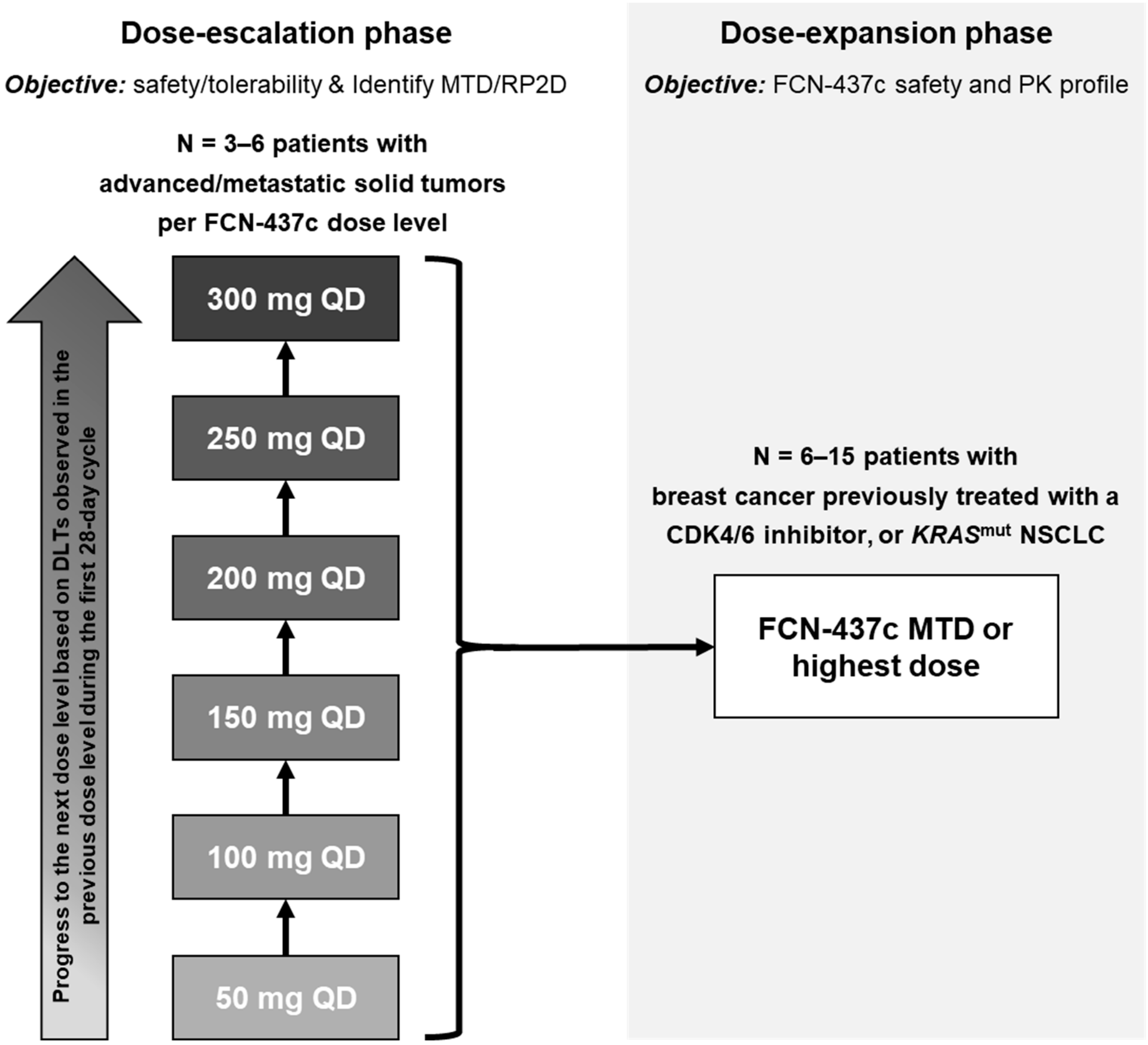

Rationale underlying the selection of tumors to be tested

Dose-escalation phase: advanced solid tumors irrespective of tumor type

Mutations in genes encoding components of the cell cycle control pathway that can lead to cyclin-dependent kinase (CDK) hyperactivation are common among solid tumors, rendering them candidates for CDK4/6 inhibition [3-5].

Dose-expansion phase: HR$^+$/HER2$^-$ breast cancer with prior CDK4/6 inhibition or KRAS^{mut} non-small cell lung cancer

Preliminary findings from the dose-escalation cohorts included one patient with *KRAS*^{mut} non-small cell lung cancer (NSCLC) who achieved a partial response, and one patient each with *KRAS*^{mut} and breast cancer who experienced stable disease. This decision was also supported by the following:

- Patients with HR$^+$/HER2$^-$ breast cancer are known to respond to CDK4/6 inhibitors (as evidenced by regulatory approval of three CDK4/6 inhibitors for this indication) [6-8]. In addition, there is evidence to suggest that patients who have previously progressed on a CDK4/6 inhibitor may benefit from retreatment or switching to a different CDK4/6 inhibitor [9-11].
- Preclinical data supported efficacy in xenograft models of breast cancer (among others) [5].
- Preclinical data suggest the existence of a synthetically lethal relationship between *KRAS* oncogenes and loss of CDK4; therefore, selective inhibition of CDK4 in *KRAS*-mutation-driven cancers such as NSCLC may induce tumor cell death [12,13].

Table S2. Causality of adverse events.

Category	Definition
**Unrelated**	Adverse events that are clearly and incontrovertibly due to extraneous causes (eg, disease, environment)
**Possibly related**	Adverse events for which a connection with the test drug administration appears unlikely but cannot be ruled out with certainty. An adverse event may be considered possibly related if or when it meets two of the following criteria:<br>1. It follows a reasonable temporal sequence from administration of the drug<br>2. It could not readily have been produced by the participant's clinical state, environmental or toxic factors, or other modes of therapy administered to the participant<br>3. It follows a known pattern of response to the test drug.
**Probably related**	Adverse events that the investigator feels with a high degree of certainty are related to the test drug. An adverse event may be considered probably related if or when it meets three of the following criteria:<br>1. It follows a reasonable temporal sequence from administration of the drug<br>2. It could not be reasonably explained by the known characteristics of the participant's clinical state, environmental or toxic factors, or other modes of therapy administered to the participant<br>3. It disappears or decreases on cessation or reduction of dose<br>– Exceptions are when an adverse event does not disappear upon discontinuation of the drug, yet drug-relatedness clearly exists (eg, as in bone marrow depression, fixed drug eruptions, or tardive dyskinesia)<br>4. It follows a known pattern of response to the test drug.

Table S3. List of pharmacokinetic parameters evaluated and their definitions.

PK parameter (unit)	Definition
**$AUC_{0–24}$** (ng·h/mL)	Area under the plasma concentration versus time curve from time 0 to 24 hours post dose
**$AUC_{0–inf}$** (ng·h/mL)	Area under the plasma concentration versus time curve from time 0 to infinity
**$AUC_{0–last}$** (ng·h/mL)	Area under the plasma concentration versus time curve from time 0 to the time of last measurable concentration
**AUC_{tau}** (ng·h/mL)	Area under the plasma concentration versus time curve during a dosing interval
**C_{max}** (ng/mL)	Maximum (peak) plasma concentration of FCN-437c
**C_{trough}** (ng/mL)	Trough plasma concentration of FCN-437c
**CL/F** (L/h)	Apparent clearance of FCN-437c
**T_{max}** (h)	Time to reach maximum (peak) plasma concentration following FCN-437c administration at steady state
**V_{ss}** (L)	Apparent volume of distribution at steady state
**V_z/F** (L)	Apparent volume of distribution
**$t_{½}$** (h)	Elimination half-life

Supplementary Results

Table S4. Most frequently observed (in ≥2 participants in the safety analysis set) TEAEs considered by the investigator to be possibly or probably related to FCN-437c and SAEs by dose group and for all participants (safety population, *N*=22).

	**Dose-escalation phase (*N*=15)**				**Dose-expansion phase (*N*=7)**		**Total (*N*=22)**
	**50 mg (*n*=3)**	**100 mg (*n*=3)**	**150 mg (*n*=3)**	**200 mg (*n*=6)**	**100 mg (*n*=3)**	**150 mg (*n*=4)**	
**TEAEs related to FCN-437c, n** (%)	**3 (100)**	**3 (100)**	**3 (100)**	**6 (100)**	**1 (33.3)**	**4 (100)**	**20 (90.9)**
Neutrophil count decreased	1 (33.3)	1 (33.3)	0	4 (66.7)	0	3 (75.0)	9 (40.9)
WBC decreased	0	1 (33.3)	2 (66.7)	3 (50.0)	0	2 (50.0)	8 (36.4)
Fatigue	1 (33.3)	0	0	4 (66.7)	0	1 (25.0)	6 (27.3)
Lymphocyte count decreased	0	0	0	2 (33.3)	1 (33.3)	2 (50.0)	5 (22.7)
Diarrhea	0	1 (33.3)	0	2 (33.3)	0	1 (25.0)	4 (18.2)
Platelet count decreased	0	0	1 (33.3)	1 (16.7)	0	2 (50.0)	4 (18.2)
Anemia	1 (33.3)	0	0	2 (33.3)	0	0	3 (13.6)
Nausea	0	1 (33.3)	0	2 (33.3)	0	0	3 (13.6)
Neutropenia	0	0	0	2 (33.3)	0	0	2 (9.1)
Taste disorder	1 (33.3)	0	1 (33.3)	0	0	0	2 (9.1)
Maculopapular rash	0	1 (33.3)	0	0	0	1 (25.0)	2 (9.1)
**SAEs, n (%)**	**2 (66.7)**	**2 (66.7)**	**3 (100)**	**4 (66.7)**	**1 (33.3)**	**4 (100)**	**16 (72.7)**
Neutrophil count decreased	1 (33.3)	0	0	3 (50.0)	0	2 (50.0)	6 (27.3)
WBC decreased	0	1 (33.3)	2 (66.7)	2 (33.3)	0	1 (25.0)	6 (27.3)
Lymphocyte count decreased	0	0	0	1 (16.7)	1 (33.3)	2 (50.0)	4 (18.2)
Anemia	1 (33.3)	0	0	1 (16.7)	0	0	2 (9.1)

Abbreviations: SAE, serious adverse event; TEAE, treatment-emergent adverse event; WBC, white blood cell.

Table S5. TEAEs leading to dose interruption that were considered by the investigator to be possibly or probably related to FCN-437c (TRAEs), by dose group and for all participants (safety population, *N*=22).

TRAEs leading to dose interruption, n (%)	Dose-escalation phase (*N*=15)				Dose-expansion phase (*N*=7)		Total
	50 mg (*n*=3)	100 mg (*n*=3)	150 mg (*n*=3)	200 mg (*n*=6)	100 mg (*n*=3)	150 mg (*n*=4)	(*N*=22)
Neutrophil count decreased	1 (33.3)	0	0	3 (50.0)	0	1 (33.3)	5 (22.7)[a]
WBC decreased	0	0	1 (33.3)	2 (33.3)	0	1 (33.3)	4 (18.2)[a]
Platelet count decreased	0	0	1 (33.3)	1 (16.7)	0	1 (33.3)	3 (13.6)[b]
Worsening fatigue	0	0	0	2 (33.3)	0	0	2 (9.1)[b]
Anemia	0	0	0	1 (16.7)	0	0	1 (4.5)[a]
Neutropenia	0	0	0	1 (16.7)	0	0	1 (4.5)[a]
Maculopapular rash	0	0	0	0	0	1 (33.3)	1 (4.5)[a]
Thrombocytopenia	0	0	1 (33.3)	0	0	0	1 (4.5)[a]
Upper respiratory tract infection	0	0	1 (33.3)	0	0	0	1 (4.5)[b]

Abbreviations: TRAE, treatment-related adverse event; WBC, white blood cell.

[a]Grade 3 except for one participant in the dose-escalation 150-mg dose cohort with grade 2 WBC decrease and another participant in the dose-escalation 200-mg dose cohort with grade 4 neutrophil count decrease and grade 4 WBC decrease. [b]Grade 2.

Table S6. Summary of key FCN-437c pharmacokinetic parameters for Cycle 1 Day 1 and Cycle 1 Day 21 by dose group.

FCN-437c dose	Timepoint	C_{max}, ng/mL	T_{max}, h	AUC_{last}, ng·h/mL	AUC_{inf}, ng·h/mL	AUC_{tau}, ng·h/mL	$t_{½}$, h	CL/F, L/h	Vz/F, L
**50 mg**	Cycle 1 Day 1, n	3	3	3	1	–	2	1	1
	Mean ± SD or median (range)	114 ± 30.4	2.0 (2.0–2.0)	1,280 ± 308	2,550 ± NC	NC	18.0 ± 2.6	19.6 ± NC	459 ± NC
	Cycle 1 Day 21, n	3	3	3	–	3	1	3	1
	Mean ± SD or median (range)	232 ± 45.6	2.0 (2.0–2.0)	3,760 ± 739	NC	3,760 ± 793	32.2 ± NC	13.6 ± 2.4	505 ± NC
**100 mg**	Cycle 1 Day 1, n	6	6	6	3	–	4	3	3
	Mean ± SD or median (range)	426 ± 227	2.0 (2.0–4.0)	5,300 ± 2,360	7,650 ± 3,550	NC	14.5 ± 2.6	14.7 ± 5.4	275 ± 79
	Cycle 1 Day 21, n	6	6	6	–	6	5	6	5
	Mean ± SD or median (range)	830 ± 377	2.0 (2.0–4.0)	12,000 ± 5,490	NC	12,000 ± 5,490	28.7 ± 11.4	10.6 ± 6.7	477 ± 453
**150 mg**	Cycle 1 Day 1, n	7	7	7	2	–	7	2	2
	Mean ± SD or median (range)	640 ± 250	4.0 (4.0–4.0)	7200 ± 2,740	7,040 ± 2,650	NC	20.5 ± 3.8	22.9 ± 8.6	554 ± 201
	Cycle 1 Day 21, n	2	2	2	–	2	2	2	2
	Mean ± SD or median (range)	1,100 ± 68 9	4.0 (4.0–4.0)	21,600 ± 16,600	NC	21,600 ± 16,600	45.5 ± 29.3	9.8 ± 4.6	486 ± 80
**200 mg**	Cycle 1 Day 1, n	6	6	6	2	–	5	2	2
	Mean ± SD or median (range)	969 ± 340	3.0 (2.0–4.0)	12,400 ± 4,370	14,400 ± 2,300	NC	18.3 ± 3.8	14.1 ± 2.3	323 ± 139
	Cycle 1 Day 21, n	2	2	2	–	2	1	2	1
	Mean ± SD or median (range)	1,590 ± 12 7	3.0 (2.0–4.0)	23,800 ± 1,190	NC	23,800 ± 1,190	14.9 ± NC	8.4 ± 0.4	175 ± NC

Abbreviations: $AUC_{0–24}$, area under the plasma concentration versus time curve between time 0 and 24 hours post dose; AUC_{inf}, area under the plasma concentration versus time curve between time 0 and infinity; AUC_{last}, area under the plasma concentration versus time curve between time 0 and the last measurable plasma concentration; AUC_{tau}, area under the plasma concentration versus time curve for a dosing interval; CL/F, apparent clearance of FCN-437c; C_{max}, maximum plasma concentration; NC, not calculable; SD, standard deviation; $t_{½}$, elimination half-life; T_{max}, time to maximum (peak) plasma concentration following FCN-437c administration at steady state; Vz/F, apparent volume of distribution.

Supplementary References

1. Eisenhauer, E.A.; Therasse, P.; Bogaerts, J.; Schwartz, L.H.; Sargent, D.; Ford, R.; Dancey, J.; Arbuck, S.G.; Gwyther, S.; Mooney, M.M.; et al. New response evaluation criteria in solid tumours: Revised RECIST guideline (version 1.1). *Eur. J. Cancer* **2009**, *45*, 228-247, doi:10.1016/j.ejca.2008.10.026.
2. National Cancer Institute (U. S.). Common Terminology Criteria for Adverse Events (CTCAE) Version 5.0. **2017**.
3. Du, Q.; Guo, X.; Wang, M.; Li, Y.; Sun, X.; Li, Q. The application and prospect of CDK4/6 inhibitors in malignant solid tumors. *J. Hematol. Oncol.* **2020**, *13*, 41, doi:10.1186/s13045-020-00880-8.
4. Hamilton, E.; Infante, J.R. Targeting CDK4/6 in patients with cancer. *Cancer Treat. Rev.* **2016**, *45*, 129-138, doi:10.1016/j.ctrv.2016.03.002.
5. Schettini, F.; De Santo, I.; Rea, C.G.; De Placido, P.; Formisano, L.; Giuliano, M.; Arpino, G.; De Laurentiis, M.; Puglisi, F.; De Placido, S.; et al. CDK 4/6 inhibitors as single agent in advanced solid tumors. *Front. Oncol.* **2018**, *8*, 608, doi:10.3389/fonc.2018.00608.
6. Pfizer Inc. *IBRANCE US Prescribing Information*. **2019**.
7. Novartis Pharmaceuticals Corp. *KISQALI US Prescribing Information*. **2022**.
8. Eli Lilly Co. *VERZENIO US Prescribing Information*. **2021**.
9. Bardia, A.; Hurvitz, S.A.; DeMichele, A.; Clark, A.S.; Zelnak, A.; Yardley, D.A.; Karuturi, M.; Sanft, T.; Blau, S.; Hart, L.; et al. Phase I/II trial of exemestane, ribociclib, and everolimus in women with $HR^+/HER2^-$ advanced breast cancer after progression on CDK4/6 inhibitors (TRINITI-1). *Clin. Cancer Res.* **2021**, *27*, 4177-4185, doi:10.1158/1078-0432.CCR-20-2114.
10. Wander, S.A.; Han, H.S.; Zangardi, M.L.; Niemierko, A.; Mariotti, V.; Kim, L.S.L.; Xi, J.; Pandey, A.; Dunne, S.; Nasrazadani, A.; et al. Clinical outcomes with abemaciclib after prior CDK4/6 inhibitor

progression in breast cancer: a multicenter experience. *J. Natl. Compr. Canc. Netw.* **2021**, 1-8, doi:10.6004/jnccn.2020.7662.

11. dos Anjos, C.H.; Razavi, P.; Herbert, J.; Colon, J.; Gill, K.; Modi, S.; Bromberg, J.; Dang, C.T.; Liu, D.; Norton, L.; et al. A large retrospective analysis of CDK 4/6 inhibitor retreatment in ER+ metastatic breast cancer (MBC). *J. Clin. Oncol.* **2019**, *37*, 1053-1053, doi:10.1200/JCO.2019.37.15_suppl.1053.
12. Li, K.; You, J.; Wu, Q.; Meng, W.; He, Q.; Yang, B.; Zhu, C.; Cao, J. Cyclin-dependent kinases-based synthetic lethality: Evidence, concept, and strategy. *Acta Pharm. Sin. B* **2021**, *11*, 2738-2748, doi:10.1016/j.apsb.2021.01.002.
13. Puyol, M.; Martin, A.; Dubus, P.; Mulero, F.; Pizcueta, P.; Khan, G.; Guerra, C.; Santamaria, D.; Barbacid, M. A synthetic lethal interaction between K-Ras oncogenes and Cdk4 unveils a therapeutic strategy for non-small cell lung carcinoma. *Cancer Cell* **2010**, *18*, 63-73, doi:10.1016/j.ccr.2010.05.025.
